# MDM4 expression as an indicator of *TP53* reactivation by combined targeting of *MDM2* and *MDM4* in cancer cells without *TP53* mutation

**DOI:** 10.18632/oncoscience.103

**Published:** 2014-11-25

**Authors:** Mitsuaki Hirose, Kenji Yamato, Shinji Endo, Rie Saito, Takunori Ueno, Sachiko Hirai, Hideo Suzuki, Masato Abei, Yukikazu Natori, Ichinosuke Hyodo

**Affiliations:** ^1^ Department of Gastroenterology and Hepatology, Institute of Clinical Medicine, Graduate School of Comprehensive Human Sciences, University of Tsukuba, Tsukuba, Ibaraki, Japan; ^2^ RNAi Company Ltd., Hongo, Bunkyo-ku, Tokyo, Japan

**Keywords:** siRNA, p53, MDM2, MDM4, knockdown, *TP53*-reactivation

## Abstract

MDM2 and MDM4, a structurally related MDM2 homolog, negatively regulates expression and functions of *TP53* tumor suppressor gene. To explore the precise expression patterns and function of *MDM2* and *MDM4* in wild-type (wt) *TP53* cancer cells, we analyzed 11 various cancer cell lines with wt *TP53*. All cell lines exhibited deregulated expression of MDM2 and MDM4, and were divided into two distinct types; the one expressing high levels of MDM4 and another expressing low levels of MDM4. The low MDM4 type expressed higher MDM2 levels than the high MDM4 type. In cells with high MDM4 expression, knockdown of *MDM4* or *MDM2* reactivated *TP53*, and simultaneous knockdown of *MDM2* and *MDM4* synergistically reactivated *TP53*. In contrast, in cells with low MDM4 expression, knockdown of only *MDM2* reactivated *TP53*. These results suggest that both *MDM2* and *MDM4* are closely involved in *TP53* inactivation in cancer cells with high MDM4 expression, whereas only *MDM2*, and not *MDM4*, is a regulator of *TP53* in cells with low MDM4 expression. MDM4 expression in wt *TP53*-tumors is a potential indicator for *TP53* reactivation cancer therapy by simultaneous targeting of *MDM4* and *MDM2*. Specific knockdown of *MDM2* and *MDM4* might be applicable for *TP53* restoration therapy.

## INTRODUCTION

The tumor suppressor protein p53 is a transcriptional factor that controls multiple genes to regulate the cell cycle, apoptosis, DNA repair, and senescence [1-4]. Approximately half of human cancers have mutations in the *TP53* gene [5], indicating that *TP53* inactivation is pivotal in cancer development. The remaining cancers retain the wild-type (wt) status of *TP53*, which is inhibited by deregulated upstream modulators and/or inactivation of downstream effectors [1, 6].

The human homolog of murine double minute 2 (MDM2) is a major negative regulator of p53 through binding to its transactivation domain, thereby resulting in subsequent suppression of transcriptional activity [7, 8]. In addition, the RING (Really Interesting New Gene) finger domain of MDM2 functions as an E3 ubiquitin ligase that mediates ubiquitin-dependent degradation of p53 [9-11]. *MDM2* is a transcriptional target of p53, forming an autoregulatory feedback loop [12, 13]. *TP53* is also negatively regulated by MDM4, an MDM2 homologue [14, 15]. Like MDM2, MDM4 represses p53 transcriptional activity by direct binding of its binding domain, which is located in the N-terminal region, to the transactivation domain of p53 [14]. Although MDM4 possess a RING finger domain, it lacks E3 ligase activity and is unable to directly decrease p53 stability [14], but rather enhances the E3 ligase activity toward p53 by forming a heterodimer with MDM2 via the RING domains of both molecules [16, 17]. MDM2 also destabilizes the structure of MDM4 via ubiquitination [18]. Both *MDM2* and *MDM4* function as oncogenes and their deregulated expression has been reported in various types of human cancers, including soft tissue sarcoma, breast cancer, retinoblastoma, and melanoma [19-23]. However, to date, the expression patterns and functional roles of *MDM2* and *MDM4* in cancer cells with or without *TP53* mutations remain uncertain.

Restoration of wt *TP53* function in tumors leads to rapid tumor regression by induction of apoptosis or senescence and can be applicable to cancer treatment [19]. Several small molecular inhibitors of the interactions between MDM2 and p53 have been shown to restore *TP53* activity in tumors expressing high MDM2 levels [24-26]. Similarly, MDM4 antagonists have been reported. Among them, SAH-p53-8 binds and inhibits more efficiently to MDM4 than to MDM2 and exerts antitumor effects in cancer cells expressing high MDM4 levels [21, 27].

Synthetic small interfering RNAs (siRNAs) are not only a powerful tool for functional gene analysis [28, 29], but has been intensively explored for application to therapy of human cancer and other diseases with some promising results [30-32]. siRNAs often silence the expression of untargeted genes with partial sequence complementarities (off-target effects) [33, 34]. However, such nonspecific effects can be avoided by DNA replacement in the seed region of the guide strand (first 6–8 bases from the 5′ end) and the complementary sequences of the passenger strand, which has been designated as a double-stranded RNA–DNA chimera (dsRDC) [35]. Considering the recent progress in RNAi technology, synthetic siRNAs targeting *MDM2* and *MDM4* may present an alternative mechanism to induce *TP53* restoration.

In the present study, we carefully analyzed MDM2 and MDM4 expression levels in various cancer cell lines with and without *TP53* mutations and found that MDM2 and MDM4 were deregulated in all wt *TP53* cancer cells. To probe the roles of *MDM2* and *MDM4* in *TP53* regulation in cancer cells, we selected efficient and specific dsRDC-modified siRNAs targeting *MDM2* and *MDM4*. Individual and combined knockdown of *MDM2* and *MDM4* revealed their roles in *TP53* inactivation in wt *TP53* cancer cells with different patterns of MDM2 and MDM4 expression, which provided us with a rationale for the selection of *MDM2* and *MDM4* as targets in *TP53* restoration therapy of cancers.

## RESULTS

### Expression levels of MDM2 and MDM4 in cancer cell lines

**Figure 1:**
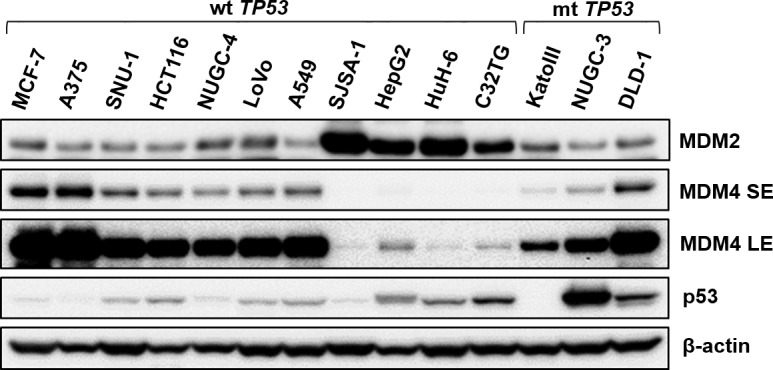
Expression levels of p53, MDM2, and MDM4 in cancer cell lines Expression levels of p53, MDM2, and MDM4 were examined in 14 cancer cell lines (11 wt *TP53* cell lines and 3 mt *TP53* cell lines) by immunoblotting. SE, short exposure; LE, long exposure.

We examined the expression levels of MDM2 and MDM4 in 14 cancer cell lines including 11 wt *TP53* and three mutant (mt) *TP53* cell lines by immunoblotting (Figure 1). wt *TP53* cell lines were divided into two groups according to levels of MDM4: seven cell lines (MCF-7, A375, SNU-1, HCT116, NUGC-4, LoVo, and A549) expressed high levels of MDM4, whereas the remaining four cell lines (SJSA-1, HepG2, HuH-6, and C32TG) expressed low levels of MDM4. Interestingly, all cell lines expressing low MDM4 levels accumulated higher levels of MDM2 than those expressing high MDM4 levels. Cell lines carrying mt *TP53* (KATOIII, NUGC-3, and DLD-1) expressed various levels of MDM4 and MDM2. p53 was not detected in KATOIII cells, which harbored gross deletions of both *TP53* alleles.

### Efficient siRNAs targeting MDM2 and their DNA-modified forms

**Figure 2:**
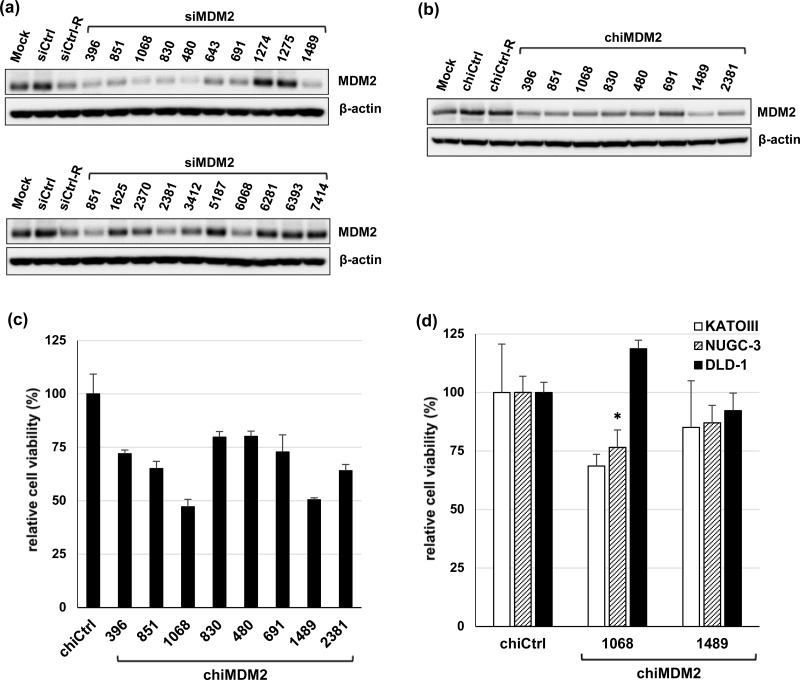
Effects of siRNAs targeting MDM2 and their dsRDC forms on MDM2 expression and cell growth (a) Two previously reported (396 and 851) and 17 new siMDM2s were analyzed for their effects on MDM2 expression in SJSA-1 cells by immunoblotting. SJSA-1 cells were transfected with mock, control siRNA (siCtrl), control-R siRNA (siCtrl-R), and siMDM2s at 1 nM for 48 h and then examined for MDM2 expression by immunoblotting. (b) Control siRNA, control-R siRNA, and eight effective siMDM2s, including two previously reported and six new were converted to dsRDC forms (chiCtrl, chiCtrl-R, and chiMDM2s), and examined for MDM2 knockdown activity in SJSA-1 cells 48 h after transfection at 1 nM. (c) Effect of chiMDM2s on growth of SJSA-1 cells were examined. The cells were transfected with control dsRDC (chiCtrl) or eight chiMDM2s at 1 nM for 5 days and then assayed for relative viable cell number using the WST-8 assay (mean ± SD; n = 3). (d) The effects of two highly effective chiMDM2s (1068 and 1489) on growth of mt *TP53*-cancer cells (KATOIII, NUCG-3, and DLD-1) after transfection at 1 nM for 5 days were examined using the WST-8 assay. Viable cell numbers relative to those transfected with control dsRDC (chiCtrl) are shown (mean ± SD; n = 3; *p < 0.05; Dunnett's test).

Seventeen new siRNAs targeting human *MDM2* transcript variant 1 (NM_002392.4) were selected using siDirect software (Supplementary Table 1) [36]. These siRNAs contained at least three mismatched base pairs in both the guide and passenger strands with a non-redundant sequence set of human genes to minimize off-target effects [37]. siRNA sequences containing single-nucleotide polymorphisms were also excluded to avoid individual differences in response. These new siRNAs targeting *MDM2* (siMDM2) and nine previously reported siMDM2s were synthesized and tested for knockdown efficiency by transfection into SJSA-1 cells and subsequent immunoblot analysis (Figure 2a) (Supplementary Figure 1). Six new (1068, 830, 480, 691, 1489, and 2381) and two previously reported siMDM2s (396 and 851) strongly suppressed MDM2 expression. These siMDM2s were converted to dsRDCs with the aim to further reduce off-target effects by decreasing the free energy of pairing stability between the seed region and off-target mRNAs [35]. As shown in Figure 2b, all dsRDC-modified siMDM2s (chiMDM2) were able to silence MDM2 expression with the most efficient silencing achieved by chiMDM2-1489. Quantitative reverse transcription (qRT)-PCR analysis demonstrated the ability of these chiMDM2s to knockdown mRNA to the same or a slightly reduced extent as compared with cognate siRNAs (Supplementary Figure 2).

The effect of chiMDM2s on the growth of cancer cells with high MDM2 expression was examined. SJSA-1 cells were transfected with chiMDM2s at 1 nM for 5 days and then subjected to the WST-8 (2-(2-methoxy-4-nitrophenyl)-3-(4-nitrophenyl)-5-(2,4-disulfophenyl)-2H-tetrazolium) cell proliferation assay. As shown in Figure 2c, most of the chiMDM2s suppressed the growth of SJSA-1 cells in proportion to the individual *MDM2* knockdown efficiency, with the exception of chiMDM2-1068, which suppressed cell growth to a greater extent than chiMDM2-1489, although the *MDM2* knockdown efficiency was inversed, suggesting that chiMDM2-1068 partially exerted MDM2–p53-indepndent growth suppression. Therefore, these two chiMDM2s were further analyzed for growth suppression of cancer cells carrying mt *TP53* (KATO III, NUGC-3, and DLD-1) (Figure 2d). chiMDM2-1489 and chiMDM2-1068 exhibited negligible effects on these cells, with the exception of chiMDM2-1068-mediated suppression of NUGC-3 cell growth.

### Selection of siRNAs targeting MDM4 and their DNA-modified forms

**Figure 3:**
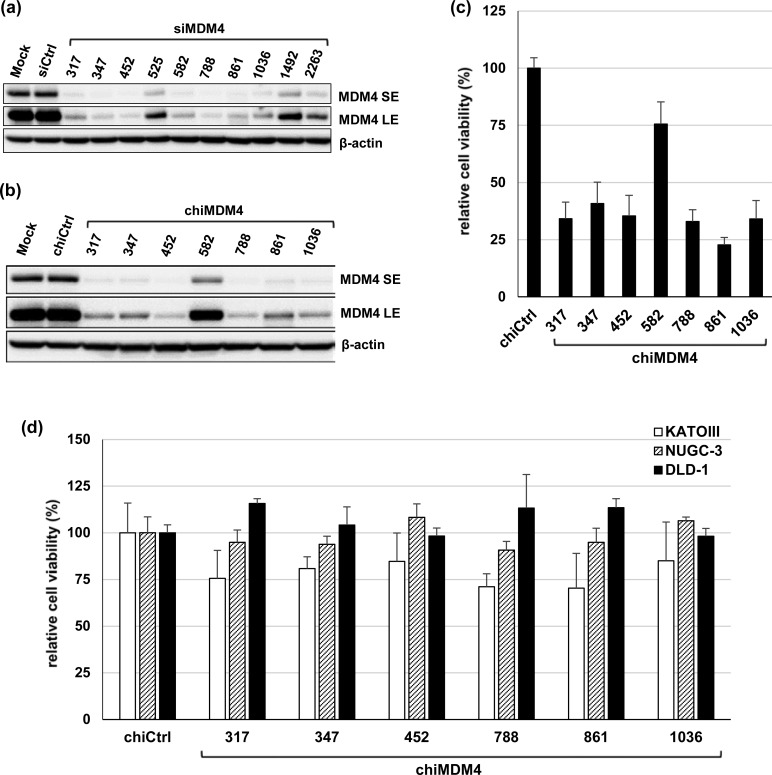
Effects of siRNAs targeting MDM4 and their dsRDC forms on MDM4 expression and cell growth (a) The effects of siMDM4s on MDM4 expression were analyzed in MCF-7 cells by immunoblotting 48 h after transfection with mock, control siRNA (siCtrl), or 10 siMDM4s at 1 nM. SE, short exposure; LE, long exposure. (b) Seven effective siMDM4s and a control siRNA were converted to dsRDC forms (chiMDM4s and chiCtrl), and analyzed for MDM4 knockdown in MCF-7 cells 48 h after transfection at 1 nM. (c) The effect of chiMDM4s on the growth of MCF-7 cells was examined. The cells were transfected with control dsRDC (chiCtrl) or seven chiMDM4s at 1 nM for 5 days and then assayed for relative viable cell number using the WST-8 assay (mean ± SD; n = 3). (d) The effects of six highly effective chiMDM4s on the growth of mt *TP53*-cancer cells (KATOIII, NUCG-3, and DLD-1) after transfecting at 1 nM for 5 days were examined using the WST-8 assay. Viable cell numbers relative to those transfected with control dsRDC (chiCtrl) are shown (mean ± SD.; n = 3; *p < 0.05; Dunnett's test).

siRNAs targeting the coding region of human *MDM4* transcript variant 1 (NM_002393.4) were similarly selected as those targeting *MDM2*. Ten new *MDM4* siRNAs (siMDM4) (Supplementary Table 1) were examined for *MDM4* knockdown efficiency in MCF-7 cells, which exhibit high levels of MDM4 expression, by immunoblot analysis (Figure 3a). Seven siMDM4s (317, 347, 452, 582, 788, 861, and 1036) showed strong suppression of MDM4 expression and were converted to dsRDCs (chiMDM4). Six chiMDM4s (317, 347, 452, 788, 861, and 1036) knocked down MDM4 expression in MCF-7 cells as efficiently as their cognate siRNAs (Figure 3b). Among these six chiMDM4s, chiMDM4-452 exhibited the highest silencing activity. qRT-PCR analysis confirmed efficient *MDM4* knockdown by each of these chiMDM4s (Supplementary Figure 3). The effect on viability of MCF-7 cells by these chiMDM4s was also tested using the WST-8 assay (Figure 3c). All chiMDM4s induced growth suppression in parallel to the *MDM4* knockdown efficiency of each. In fact, potent growth suppression was observed with most chiMDM4s (317, 347, 452, 788, 861, and 1036). chiMDM4-582 exhibited less efficient *MDM4* silencing and growth inhibitory activities of MCF-7 cells than other chiMDM4s.

Next, the effect of each chiMDM4 on the growth of mt *TP53* cancer cells was evaluated. Six effective chiMDM4s were introduced into three mt *TP53* cancer cell lines (KATO III, NUGC-3, and DLD-1) and examined for effects on cell growth suppression using the WST-8 assay. As shown in Figure 3d, chiMDM4-452 and -1036 showed negligible growth suppression, whereas other chiMDM4s exhibited mild growth suppression, but without any statistical differences.

### Effects of MDM4 and MDM2 knockdown on growth of wt TP53 cancer cells

**Figure 4:**
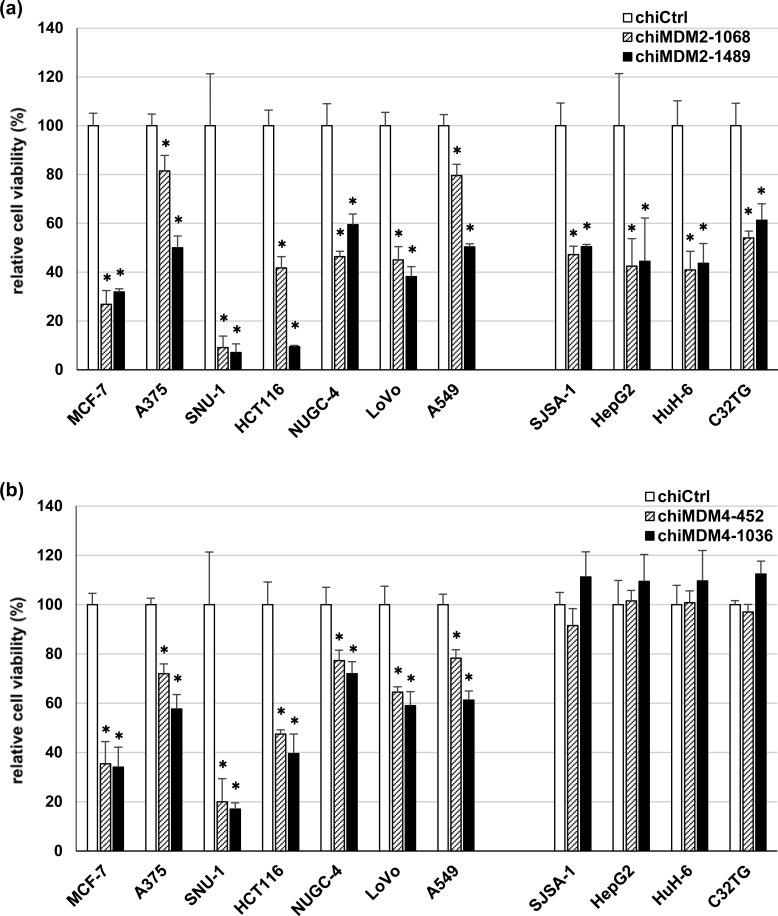
Effect of *MDM2* and *MDM4* knockdown on the growth of wt *TP53* cell lines dsRDCs targeting *MDM2* (chiMDM2-1068 and chiMDM2-1489) (a), *MDM4* (chiMDM4-452 and chiMDM4-1036) (b) and control dsRDC (chiCtrl) were transfected into seven cell lines with high MDM4 expression levels (MCF-7, A375, SNU-1, HCT116, NUCG-4, LoVo, and A549) and four cell lines with low MDM4 expression levels (SJSA-1, HepG2, HuH-6, and C32TG) at 1 nM. Five days after transfection, cell viability was determined using the WST-8 assay. Viable cell numbers relative to those transfected with chiCtrl are shown (mean ± SD; n = 3; *p < 0.05; Dunnett's test).

To examine the effect of *MDM4* and *MDM2* knockdown on the growth of wt *TP53* cancer cells, we tested the 11 previously mentioned wt *TP53* cancer cell lines, which included seven with high levels of MDM4 expression (MCF-7, A375, SNU-1, HCT116, NUGC-4, LoVo, and A549) and four with low levels of MDM4 expression (SJSA-1, HepG2, HuH-6, and C32TG). To knock down *MDM2* and *MDM4*, two dsRDCs were chosen for each target (chiMDM2-1068/-1489 and chiMDM4-452/-1036). As shown in Figure 4, each chiMDM2 inhibited growth of all wt *TP53* cancer cells regardless of the expression levels of MDM2 or MDM4, whereas chiMDM4 only suppressed the growth of cells with high MDM4 expression and not of those with low MDM4 expression (Figure 4).

**Figure 5:**
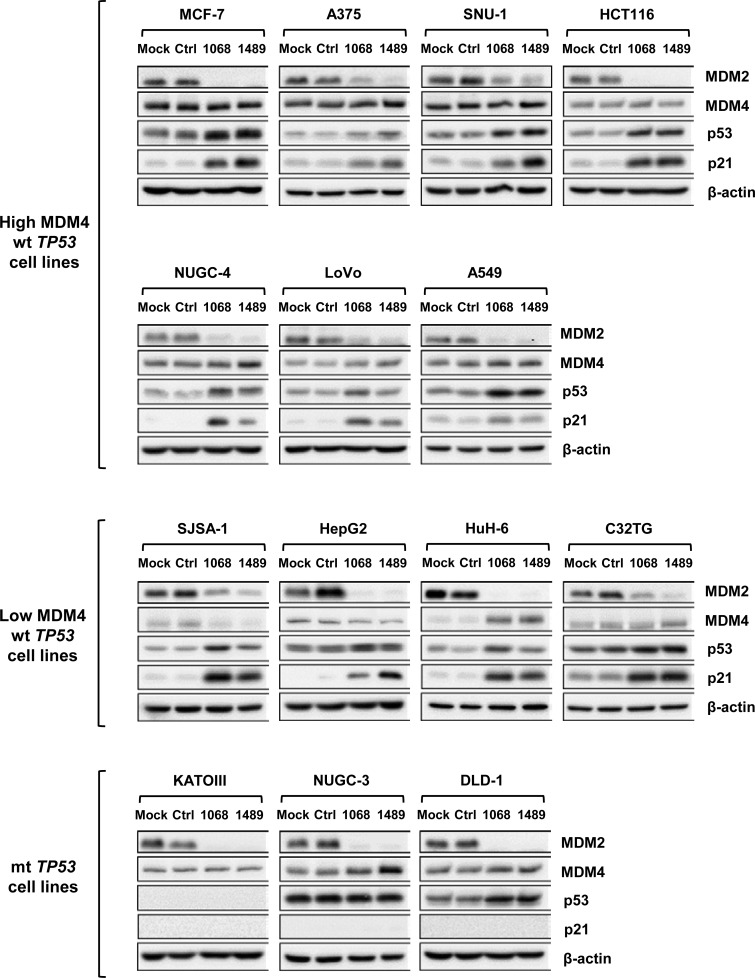
Effect of *MDM2* knockdown on expression levels of MDM2, MDM4, p53 and p21 Mock, control dsRDC (Ctrl), and two dsRDCs targeting *MDM2* (chiMDM2-1068, chiMDM2-1489) were transfected into seven cells lines with high MDM4 expression, four cell lines with low MDM4 expression and three mt *TP53* cell lines at 1 nM. Expression levels of MDM2, MDM4, p53, and p21 were analyzed by immunoblotting 2 days after transfection.

**Figure 6:**
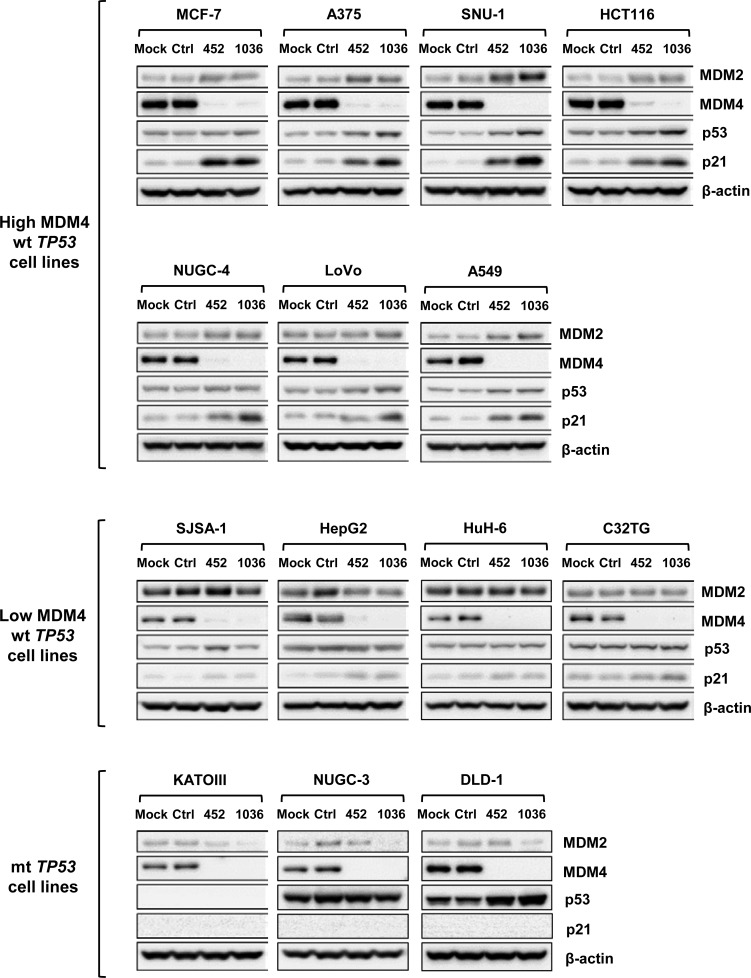
Effect of *MDM4* knockdown on expression levels of MDM4, MDM2, p53, and p21 Mock, control dsRDC (Ctrl), and two dsRDCs targeting *MDM4* (chiMDM4-452 and -1036) were transfected into seven cell lines with high MDM4 expression, four cell lines with high MDM2 expression and three mt *TP53* cell lines at 1 nM. Expression levels of MDM2, MDM4, p53, and p21 were analyzed by immunoblotting 2 days after transfection.

Next, we examined the effects of *MDM2* and *MDM4* knockdown on expression levels of p53 and p21^Waf1/Cip1^ (p21), a *TP53* responsive gene product [38], by immunoblotting. As shown in Figure 5, *MDM2* suppression increased levels of p53 and p21 in all wt *TP53* cells. MDM4 slightly accumulated in most of the wt *TP53* cells after *MDM2* knockdown, with the exception of SJSA-1 and HepG2 cells. As shown in Figure 6, in all cells with high MDM4 expression, *MDM4* knockdown slightly increased p53 levels in association with the induction of p21 and MDM2, which are known *TP53*-responsive genes, but had negligible effects on p53, p21, and MDM2 in cells with low MDM4 expression.

The effects of *MDM2* and *MDM4* knockdown on p53 and MDM4 varied among three mt *TP53* cell lines. As shown in Figures 5, *MDM2* knockdown induced mild MDM4 accumulation in NUGC-3 and DLD-1 cells, but not KATOIII cells. As shown in Figure 6, *MDM4* knockdown reduced MDM2 levels in all three mt *TP53* cell lines. Accumulation of p53 occurred in DLD-1 cells, which expressed low levels of mt p53, but not in NUGC-3 cells, which expressed high levels of mt p53. The induction of p21 did not occur in any of these mt *TP53* cell lines in response to *MDM2* or *MDM4* knockdown.

### Effects of MDM2/MDM4 double knockdown on growth of wt TP53 cancer cells

**Figure 7:**
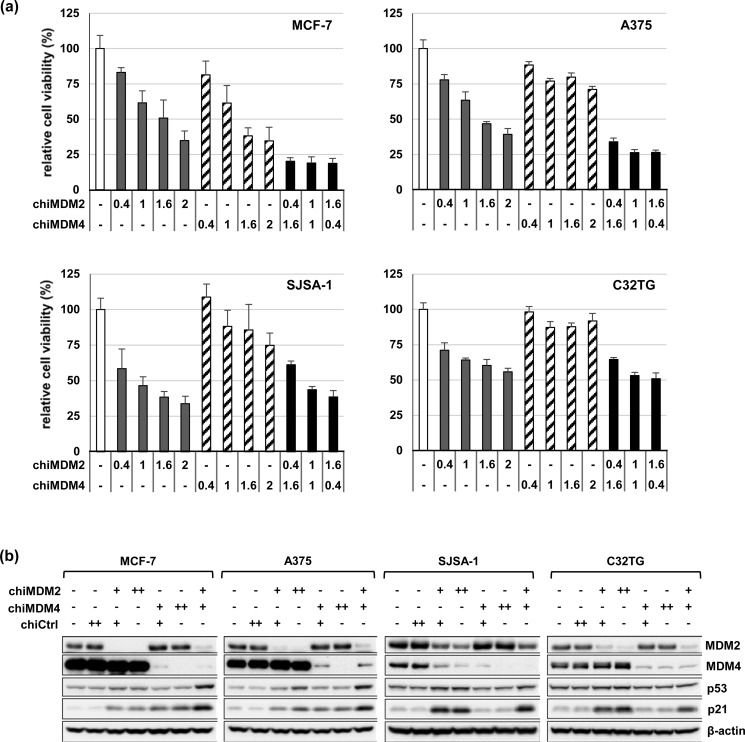
Combined knockdown of *MDM2* and *MDM4* in wt *TP53* cell lines with high and low MDM4 expression Effects of individual and simultaneous knockdown of *MDM2* and *MDM4* on cell growth (a) and expression of p53 and p21 (b) were examined in two cell lines with high MDM4 expression (MCF-7 and A375) and two cell lines with low MDM4 expression (SJSA-1 and C32TG). Cells were transfected with *MDM4* dsRDC (chiMDM4-452) alone, *MDM2* dsRDC (chiMDM2-1489) alone, or both. The total amount of dsRDCs was adjusted to 2 nM by adding control dsRDC (chiCtrl). Cell viability was determined 5 days after transfection using the WST-8 assay. Viable cell numbers of chiCtrl (2 nM) transfected cells was defined as 100% (mean ± SD; n = 3). Levels of MDM2, MDM4, p53, and p21 were analyzed by immunoblotting 2 days after transfection. In panel b, + and ++ indicates 1 nM and 2 nM of dsRDCs, respectively.

We examined the effect of *MDM2*/*MDM4* double knockdown on growth of wt *TP53* cells, which had high MDM4 expression, using chiMDM4-452 and chiMDM2-1489, which were the most potent and specific inhibitors of each respective target. Cells were transfected with various chiMDM4 concentrations along with control dsRDC-modified siRNA (chiCtrl) at a total dosage of 2 nM, as indicated. As shown in Figure 7a, chiMDM4 and chiMDM2 suppressed the growth of MCF-7 and A375 cells in a dose-dependent manner. When chiMDM2 and chiMDM4 were simultaneously transfected at three different ratios, more profound growth suppression was observed in these cells than transfection of either chiMDM2 or chiMDM4 alone at the same dosage, or even at a maximal dosage of 2 nM. Similar enhancement in growth suppression was observed in all cells with high MDM4 expression, including five other cell lines (SNU-1, HCT116, NUGC-4, LoVo, and A549) (Supplementary Figure 4). Combination index values at three different ratios of chiMDM2 and chiMDM4 were calculated in cells with high MDM4 expression with values ranging between 0.20 and 0.72, which showed that these dsRDCs promoted synergistic growth inhibition of tumor cells with high MDM4 expression (Table 1).

**Table 1:** Combination index of chiMDM2 and chiMDM4 in MDM4 overexpressed cancer cell lines

chiMDM2 (nM)	chiMDM4 (nM)	Combination index						
		MCF-7	A375	SNU-1	HCT116	NUGC-4	LoVo	A549
0.4	1.6	0.57	0.20	0.36	0.17	0.26	0.55	0.55
1.0	1.0	0.51	0.28	0.39	0.43	0.39	0.45	0.65
1.6	0.4	0.47	0.20	0.56	0.44	0.63	0.31	0.72

CI>1.1, antagonistic effect; CI = 0.9–1.1, additive; CI<0.9, synergistic effects and the lower value means the stronger synergistic effect.

chiMDM2, but not chiMDM4, alone dose-dependently suppressed the growth of cells with low MDM4 expression (SJSA-1 and C32TG). Further, chiMDM2-mediated growth inhibition was not enhanced by co-transfection with chiMDM4 in these cells (Figure 7a). Similar results were observed in two other cell lines with low MDM4 expression (Supplementary Figure 5). In mt *TP53* cells, chiMDM2, chiMDM4, and a combination of both failed to demonstrate any detectable growth suppression (Supplementary Figure 5).

### Effect of MDM2/MDM4 double knockdown on p53 expression

To explore the mechanism by which *MDM2*/*MDM4* double knockdown synergistically inhibited the growth of cells with high MDM4 expression, the effects of individual and simultaneous knockdown of *MDM2* and *MDM4* on p53 expression was examined in MCF-7 and A375 cells (Figure 7b). *MDM2* knockdown was associated with mild accumulation of MDM4 and p53, as well as the product of the downstream gene *p21*. *MDM4* knockdown slightly increased levels of MDM2 and p21 in these cells. A subtle increase in p53 expression was observed in chiMDM4-transfected A375 cells, but not in chiMDM4-transfected MCF-7 cells. Simultaneous knockdown by chiMDM2 (1 nM) and chiMDM4 (1 nM) induced expression of p53 and the downstream *p21* gene product more than either chiMDM2 or chiMDM4 alone at 1 or 2 nM in cell lines with high MDM4 expression (MCF-7, A375). In cells with low MDM4 expression (SJSA-1 and C32TG), chiMDM2 alone knocked down MDM2, which resulted in accumulation of both p53 and p21 (Figure 7b). However, chiMDM4 did not induce either p53 or p21 upregulation even though MDM4 was efficiently suppressed. Co-transfection of chiMDM2 and chiMDM4 induced accumulation of p53 and p21 to the same extent as chiMDM2.

## DISCUSSION

A fraction of wt *TP53* tumors expresses oncogenes, such as *MDM2* and *MDM4*, to inactivate *TP53* [19]. Precise expression patterns and the functional significance of *MDM2* and *MDM4* in wt *TP53* cancer cells remain to be clarified. In the present study, a careful analysis of cancer cell lines harboring wt and mt *TP53* demonstrated that all wt *TP53* cancer cell lines included in this study exhibited deregulated expression of MDM2 and MDM4. These cell lines were divided into just two distinct types, according to MDM4 expression levels; the one expressing MDM4 at high levels and another expressing MDM4 at low levels. MDM4 expression occurs when tumor cells have acquired *MDM4* amplification [19], activated *KRAS* mutations [39], or loss of miR-34a-mediated suppression [40]. Among seven wt *TP53* cancer cell lines with high MDM4 expression, one cell line (MCF-7) has MDM4 amplification [41]. Four cell lines (SNU-1, HCT116, LoVo, A549) harbor *KRAS* mutation [42, 43], suggesting that the deregulated expression of MDM4 may be caused by *KRAS* activation or along with miR-34a abnormality in these cell lines.

It is well established that *MDM2* and *MDM4* are ideal therapeutic targets for wt *TP53* tumors. However, to date, there is no biological rationale of whether *MDM2* or *MDM4* should be targeted in such tumors. Using wt *TP53* cell lines and DNA-modified siRNAs specific to *MDM2* and *MDM4*, we demonstrated here for the first time that knockdown of either *MDM4* or *MDM2* alone can reactivate the *TP53* pathway in cancer cells with high MDM4 expression, whereas knockdown of *MDM2*, but not *MDM4*, can reactivate wt *TP53* in the low *MDM4* cancer cells. Furthermore, simultaneous knockdown of *MDM2* and *MDM4* synergistically activated *TP53* and suppressed cell growth in the cancer cells with high MDM4 expression. Based on these results, we propose that both *MDM4* and *MDM2* are efficient therapeutic targets in wt *TP53* tumors cells with high MDM4 expression, whereas *MDM2*, but not necessarily *MDM4*, presents a possible therapeutic target in wt *TP53* cancer cells with low MDM4 expression.

We explored the mechanisms by which *MDM2*/*MDM4* double knockdown exhibited synergistic effects on *TP53* activation in tumor cells with high MDM4 expression. MDM4 is devoid of a nuclear transport signal and requires MDM2 to translocate from the cytoplasm to nucleus [44, 45]. Therefore, we assessed whether the synergistic effect of *MDM2*/*MDM4* double knockdown on *TP53* activation was mediated through the inhibition of nuclear transport of MDM4 by MDM2 (Supplementary Figure 6). We found that *MDM2* knockdown had no effect on nuclear localization of MDM4 in cells with high MDM4 expression (A375), suggesting that this nuclear localization was independent of MDM2 expression in these cells and that synergistic activation of *TP53* was not mediated by inhibition of nuclear transport of MDM4 in these cells. In cells with high MDM4 expression, *MDM4* silencing alone increased p53 expression. Because MDM4 has no intrinsic ubiquitin ligase function, but can enhance MDM2 ubiquitin ligase activity by forming a heterodimer with MDM2 [14, 17], both MDM2 and MDM4 may be involved in p53 degradation through the formation of heterodimers. Alternatively, *MDM4* knockdown alone enhances *MDM2* expression by releasing p53 transcriptional activity, which subsequently suppresses p53 [12, 14, 46]. Therefore, simultaneous knockdown of *MDM4* and *MDM2* may result in more potent activation of p53 by blocking this p53-MDM2 negative feedback than silencing MDM4 alone.

With the aim to employ synthetic siRNAs in *TP53*-restoration therapy, we carefully designed and screened siRNAs with high specificities and potencies to target either *MDM2* or *MDM4*. A series of siRNAs targeting *MDM2* and *MDM4* was designed using siDirect software, which enabled the selection of siRNA sequences with structural features compatible for the efficient loading of the guide strand into the RNA-induced silencing complex as well as a minimal number of off-target candidates from human genes [36, 47]. Among them, effective siRNAs with high knockdown efficiency were chosen by cell-transfection experiments and then converted to DNA-modified siRNAs with 6-base pair double-stranded DNA substitutions [35]. This modification offers a great advantage by lowering off-target activity by decreasing the free energy between the seed regions and off-target mRNAs and avoiding passenger strand-mediated RNAi [35, 48]. Three dsRDCs targeting *MDM2* (chiMDM2-1489, 1068, and 2381) and six targeting *MDM4* (chiMDM4-861, 452, 1036, 317, 347, and 788) showed potent silencing activity at a concentration as low as 1 nM. siRNAs interfere with the endogenous miRNA pathway by competing with molecules involved in miRNA production, such as AGO2, when introduced at high concentrations [49-51]. Intracellular concentrations of most functional miRNAs are between 3 and 100 nM [52]. To avoid disruptions to the miRNA pathway, it is necessary to introduce siRNAs with high silencing activities into cells at the lowest concentrations possible. dsRDCs targeting *MDM2* and *MDM4* selected in this study had a half maximal inhibitory concentration (IC value) of less than 1 nM and were used at 1 nM in most of our experiments. With the development of an efficient delivery system of oligonucleotides, dsRDCs targeting *MDM2* and *MDM4* could be applied to the treatment of wt *TP53* cancers.

Some earlier reports using *TP53* switchable mice with *MDM2*- and *MDM4*-deficient backgrounds showed transient restoration of *TP53* activity in normal tissues in the absence of *MDM2*, resulting in 100% fatality within 5–6 days [53], whereas *TP53* activation in the absence of *MDM4* was nonlethal and reversible [54]. These data indicate that systemic administration of MDM4 inhibitors may be better tolerated than MDM2 inhibitors, therefore, *MDM4* knockdown using our dsRDC might be a valuable therapeutic strategy for treatment of tumors with high MDM4 expression levels but no *TP53* mutations. Currently, we are exploring expression patterns of MDM2, MDM4, and p53 as well as the genotypes of various human tumor samples.

Some cancer cells expressing high levels of MDM4 are reportedly resistant to small-molecule MDM2 inhibitors [55-57]. However, our results clearly demonstrated that specific *MDM2* knockdown suppressed growth of wt *TP53* cells regardless of the expression levels of MDM2 and MDM4. The action mechanisms of small molecular inhibitors and siRNAs differ because small molecular inhibitors bind MDM2 at the p53-binding pocket and disrupt MDM2–p53 interactions and increase p53 expression, resulting in enhanced *MDM2* induction, which might dysregulate other *MDM2*-target molecules. In contrast, siRNAs targeting *MDM2* suppress only *MDM2*. This phenomenon might explain the discrepancy in our results.

Besides controlling *TP53* activity, MDM2 has been reported to regulate E2F1 transcriptional activity and expression of p21, FOXO3a, and XIAP [58-61]. MDM4 has been also reported to inhibit p21 and Smad family proteins [62, 63]. The results of the present study showed that knockdown of *MDM2* and *MDM4* by respective dsRDCs at 1 nM had no effect on growth of mt *TP53* cancer cells expressing various levels of MDM4, suggesting that growth suppression by *MDM2* and *MDM4* knockdown is entirely dependent on wt *TP53* and that suppression of *TP53*-independent activities had a minimal effect on vitro growth of tumors expressing mt *TP53*. The mild growth suppression of NUGC-3 and DLD-1 cells observed by transfection of chiMDM2-1489 at 2 nM suggested the presence of nonspecific effects, such as inhibition of miRNA generation, even though the siRNA concentration was very low.

In conclusion, we showed that most wt *TP53* cancer cells exhibited deregulation of MDM2 and MDM4. Specific knockdown of *MDM2* and *MDM4* with DNA-modified siRNAs clearly revealed the ability of *MDM2* and *MDM4* to inactivate wt *TP53* in cancer cells. The results of this study provide rationale for the selection of *MDM2* and *MDM4* as therapeutic targets in cancer cells expressing wt *TP53*. MDM4 expression in wt *TP53*-tumors is a potential indicator for *TP53* reactivation by combined *MDM4* and *MDM2*-targeted cancer therapy. Our specific and potent DNA-modified siRNAs targeting *MDM2* and *MDM4* might be applicable to *TP53* restoration therapy for human cancers.

## MATERIALS AND METHODS

### Cell lines

Fourteen tumor cell lines were used: eleven cell lines with wt *TP53* (MCF-7 breast cancer, A375 melanoma, HCT116 colon cancer, NUGC-4 gastric cancer, LoVo colon cancer, SJSA-1 osteosarcoma, HepG2 hepatocellular carcinoma, HuH-6 hepatocellular carcinoma, A549 lung cancer, and C32TG melanoma) [42, 64-66], and three cell lines with mt *TP53* (KATOIII gastric cancer, NUGC-3 gastric cancer, and DLD-1 colon cancer) [64]. The MCF-7, A375, SNU-1, HCT116, LoVo, SJSA-1, and DLD-1 cell lines were purchased from the American Type Culture Collection (Rockville, MD, USA). The NUGC-4, HepG2, and KATOIII cell lines were obtained from the Riken BioResource Center Cell Bank (Tsukuba, Japan). The NUGC-3, A549, HuH-6 and C32TG cell lines were obtained from the Japanese Collection of Research Bioresources Cell Bank (Osaka, Japan). MCF-7, SNU-1, NUGC-4, SJSA-1, KATOIII, NUGC-3, and DLD-1 cells were cultured in RPMI 1640 medium (Sigma–Aldrich, St. Louis, MO, USA) supplemented with 10% fetal bovine serum (FBS; Nichirei Biosciences, Tokyo, Japan). A375, HepG2, and HuH-6 cells were cultured in Dulbecco's modified Eagle's medium (Sigma–Aldrich) containing 10% FBS. HCT116 cells were cultured in McCoy's 5A medium (Sigma–Aldrich) with 10% FBS. LoVo cells were cultured in Ham's F12K medium (Invitrogen, Carlsbad, CA, USA) with 10% FBS.

### siRNAs and transfection

Sequences of siRNAs used in this study are summarized in Supplementary Table 1. All siRNAs targeting *MDM2* and *MDM4* were designed using siDirect software (http://sidirect2.rnai.jp), as reported previously [36]. The control siRNA was an artificial sequence designed to have all features of siRNAs inducing potent RNAi and the least homology to human and mouse genes. Control-R siRNA consisted of randomized sequences of the control siRNA. Control siRNA and complementary dsRDC-modified forms were included in all experiments [07]. siRNAs were converted to dsRDCs by substituting six ribonucleotides from the 5′ end of the guide strand and eight from the 3′ end of the passenger strand with corresponding deoxynucleotides [35, 67]. siRNA transfection was performed using Lipofectamine RNAiMAX (Invitrogen) as reported previously [67].

### Immunoblot analysis

Sodium dodecyl sulfate-polyacrylamide gel electrophoresis and immunoblot analysis were performed as previously described [68]. The primary and secondary antibodies used in this study were as follows: mouse monoclonal antibody against MDM2 (2A10) (Abcam, Cambridge, UK); rabbit polyclonal antibody against MDM4 (Bethyl Laboratories, Montogomery, TX, USA); mouse monoclonal antibodies against p21Waf1/Cip1 (DCS60) and β-actin (8H10D10) (Cell Signaling Technology, Danvers, MA, USA); and anti-TP53 mouse monoclonal antibody (BP53-12; Cell Sciences, Canton, MA, USA). Both horseradish peroxidase-conjugated anti-mouse IgG sheep and anti-rabbit IgG donkey sera were purchased from GE Healthcare (Buckinghamshire, UK). Chemiluminescent detection was performed using ECL Prime Western Blotting Detection Reagent (GE Healthare) and the Ez-Capture II Imaging System (Atto Corp., Tokyo, Japan).

### Quantitative reverse transcription (qRT)-PCR

RNA samples were extracted from cell lysate using 40 μL per well of RealTime ready Cell Lysis reagent (Roche Diagnostics, Mannheim, Germany), according to the manufacturer's instructions. cDNA was synthesized using 2 μL of RNA and 8 μL of Transcriptor Universal cDNA Master (Roche Diagnostics) in 20 μL-reactions. qRT-PCR assays were performed using Applied Biosystems 7500 Fast Real-Time PCR System (Applied Biosystems, Foster City, CA, USA) in 96-well plates. Primers and TaqMan probes for *MDM2*, *MDM4*, and *18S ribosomal RNA* (*18SrRNA*) were obtained from Applied Biosystems (Assay ID: Hs00234753, Hs00967238, and Hs03928990_g1, respectively). Reactions were performed in duplicate under standard thermocycling conditions in a 20-μL volume containing 0.8 μL of cDNA, 900 nM of primers, 250 nM of the probe, and 10 μL of TaqMan Gene Expression Master Mix (Applied Biosystems), according to the manufacturer's protocol. The amount of target mRNA was examined and normalized to that of *18S rRNA*.

### Cell viability

WST-8 colorimetric assays were performed using a Cell Counting kit-8 (Dojin Laboratories, Kumamoto, Japan) according to the manufacturer's protocol. Cells were incubated for 5 days after transfection and then analyzed using an iMark microplate reader (Bio-Rad, Hercules, CA, USA). The absorbance of the plates was read at wavelengths of 450 nm and 620 nm.

### Combination index

Quantification of chiMDM2 and chiMDM4 synergy was determined by the Chou–Talalay method for drug combination using CalcuSyn software (Biosoft, Cambridge, UK) [69]. A combination index (CI) < 0.9 indicates synergism, 09–1.1 indicates additivity, and >1.1 indicates antagonism.

### Immunofluorescence

Cells were fixed for 15 min in 4% paraformaldehyde at room temperature, and aldehydes were neutralized by soaking coverslips in phosphate-buffered saline (PBS) with 0.1% tween 20 (PBS-T) containing 50 mM glycine at room temperature. Then, the cells were permeabilized in PBS-T with 0.1% Triton X-100 solution for 15 min on ice, blocked for 60 min in PBS-T solution containing 5% normal goat serum (Vector Laboratories, Burlingame, CA, USA) (blocking solution) at room temperature, and then reacted with rabbit polyclonal antibody against MDM4 (Bethyl Laboratories) diluted with blocking solution. After overnight incubation at 4°C, the cells were reacted with fluorescein isothiocyanate-conjugated goat anti-rabbit IgG antibody (Bethyl Laboratories) diluted with washing buffer for 60 min at room temperature. Nuclei were counterstained with 4′,6-diamidino-2-phenylindole (DAPI). Confocal fluorescence images were obtained using Leica TCS SP5 confocal microscope (Leica Microsystems GmbH, Wetzlar, Germany)

### Statistical analysis

All data are expressed as the mean ± standard deviation (SD). Statistical significance of differences between various groups was evaluated using the Dunnett's test. P-values < 0.05 were considered significant.

## SUPPLEMENTARY MATERIAL FIGURES AND TABLE


