# Supplementary material for: MDM4 expression as an indicator of TP53 reactivation by combined targeting of MDM2 and MDM4 in cancer cells without TP53 mutation
Source: Oncoscience. 2014 Nov 25;1(12):830–43. doi: 10.18632/oncoscience.103 (PMC4303891; doi:10.18632/oncoscience.103)
Supplement: Supplementary file 1 [file oncoscience-01-0830-s001.pdf]

MDM4 expression as an indicator of *TP53* reactivation by combined targeting of *MDM2* and *MDM4* in cancer cells without *TP53* mutation

Supplementary Material

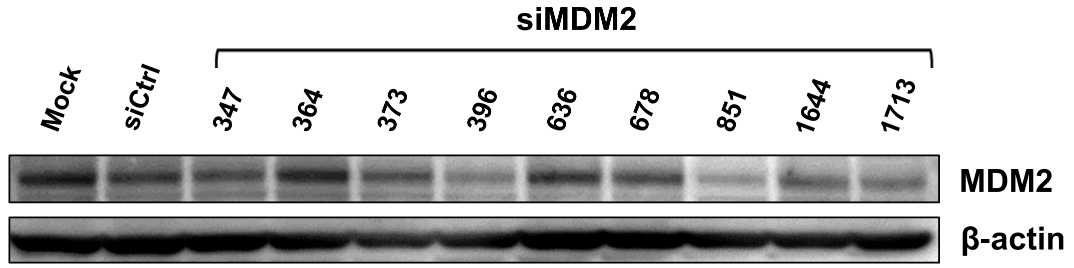

Supplementary Figure 1: Knockdown efficiency of siRNAs targeting *MDM2*. SJSA-1 cells were transfected with mock, control siRNA (siCtrl), and nine reported siMDM2s at 1 nM for 48 h and then examined for MDM2 expression by immunoblotting.

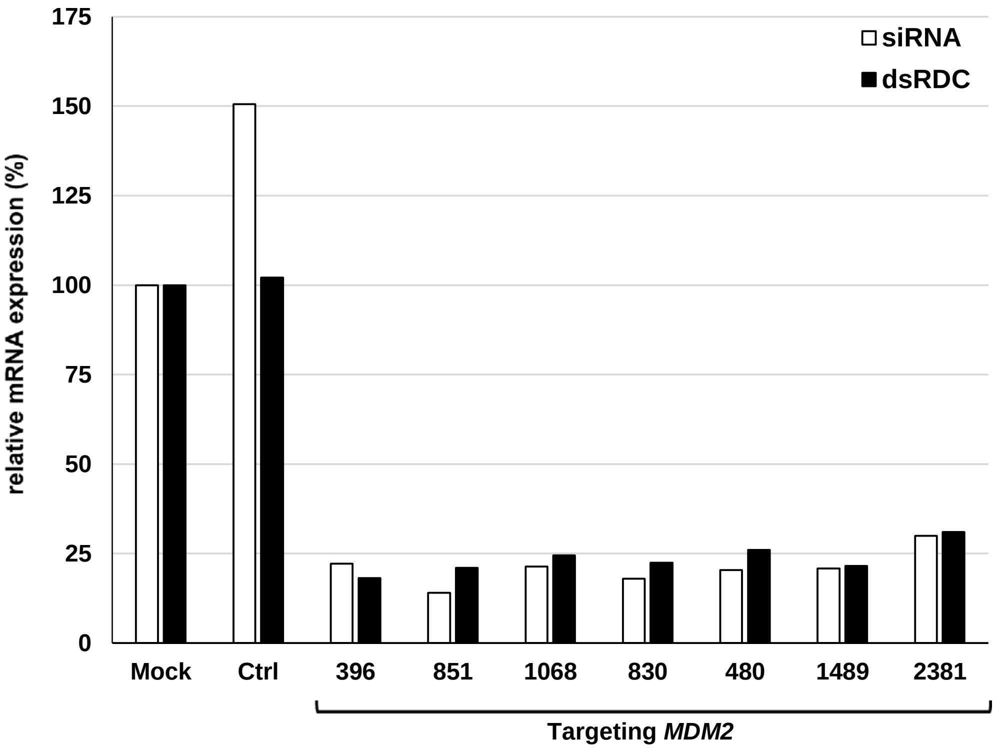

Supplementary Figure 2: Knockdown efficiency of siRNAs targeting *MDM2* and their dsRDC forms. SJSA-1 cells were transfected with mock, control (Ctrl) siRNA, seven siMDM2s, and their dsRDC forms at 1 nM for 48 h and then examined for *MDM2* mRNA expression by qRT-PCR. All experiments were performed in duplicate.

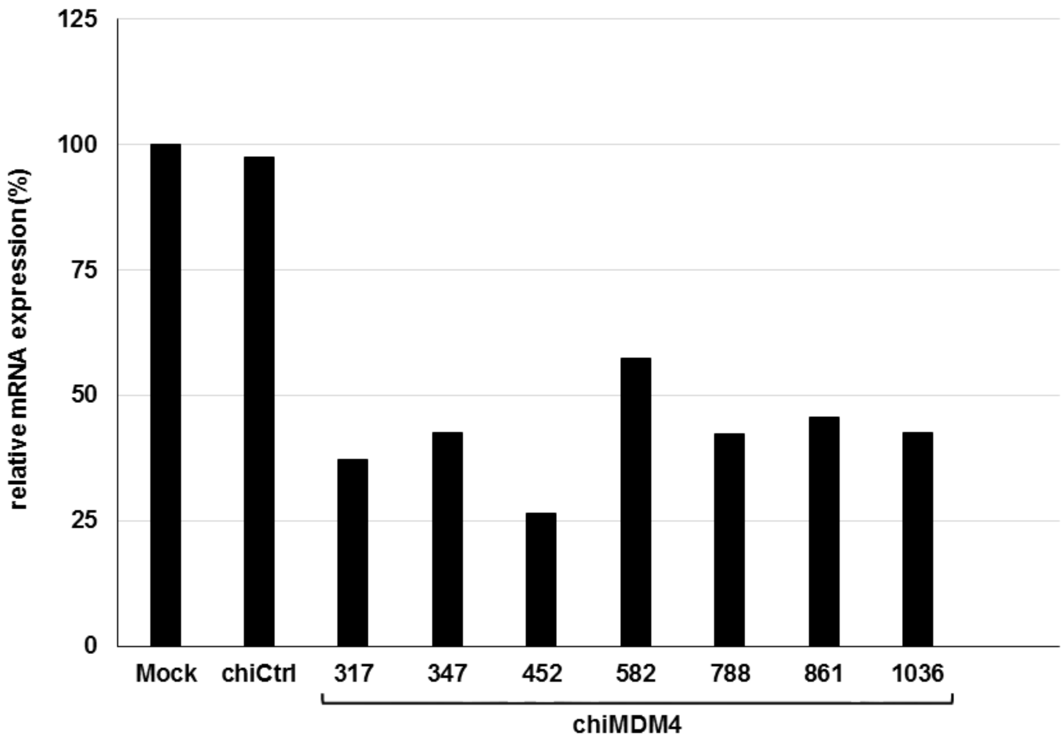

Supplementary Figure 3: Knockdown efficiency of dsRDC-modified siRNAs targeting *MDM4*. MCF-7 cells were transfected with mock, control dsRDC (chiCtrl), and seven dsRDCs targeting *MDM4* (chiMDM4) at 1 nM for 48 h and then examined for *MDM4* mRNA expression by qRT-PCR. All experiments were performed in duplicate.

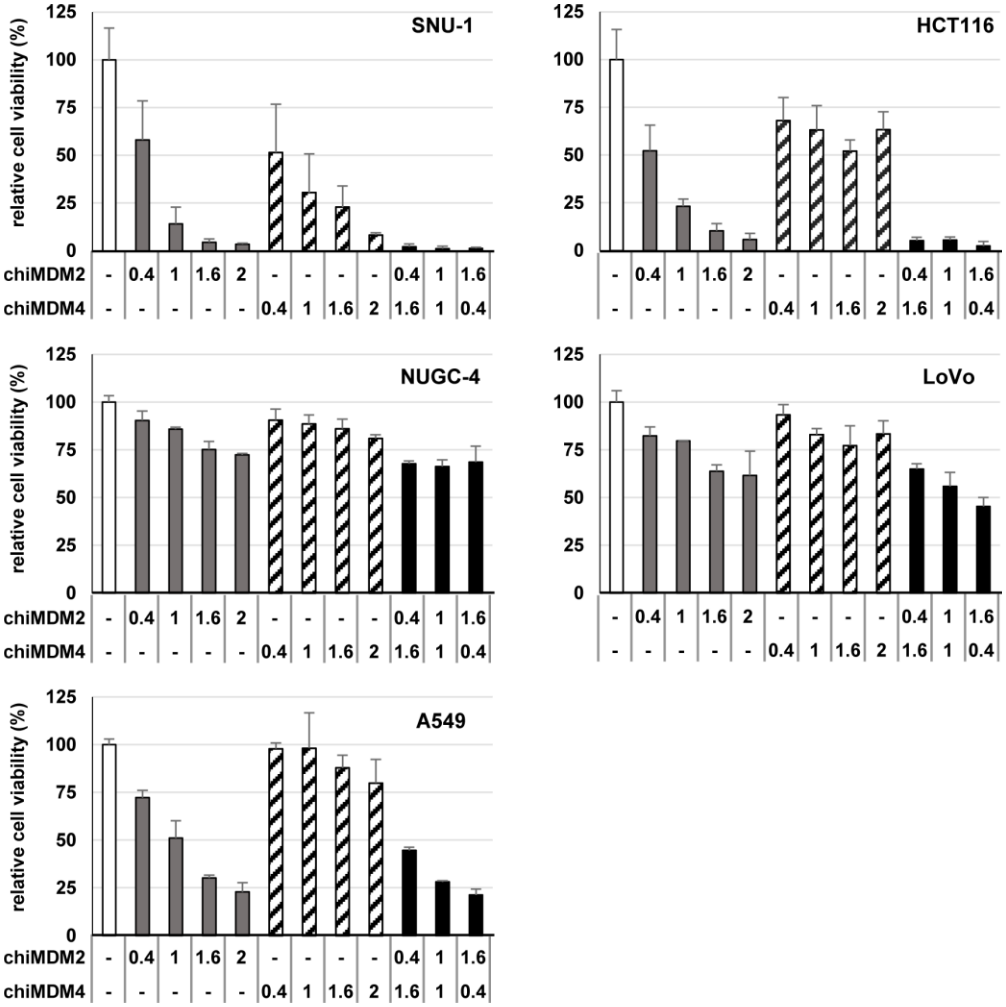

Supplementary Figure 4: Evaluation of combined knockdown of *MDM2* and *MDM4* on the growth of wtTP53 cell lines with high MDM4 expression. Effects of individual and simultaneous knockdown of *MDM2* and *MDM4* on growth were examined in five wtTP53 cell lines with high MDM4 expression. Cells were transfected with *MDM4* dsRDC (chiMDM4-452) alone, *MDM2* dsRDC (chiMDM2-1489) alone, or both. The total amount of dsRDC was adjusted to 2 nM by adding control dsRDC (chiCtrl). Cell viability was determined 5 days after transfection using the WST-8 assay. The number of viable chiCtrl (2 nM) transfected cells was defined as 100% (mean ± SD; n = 3).

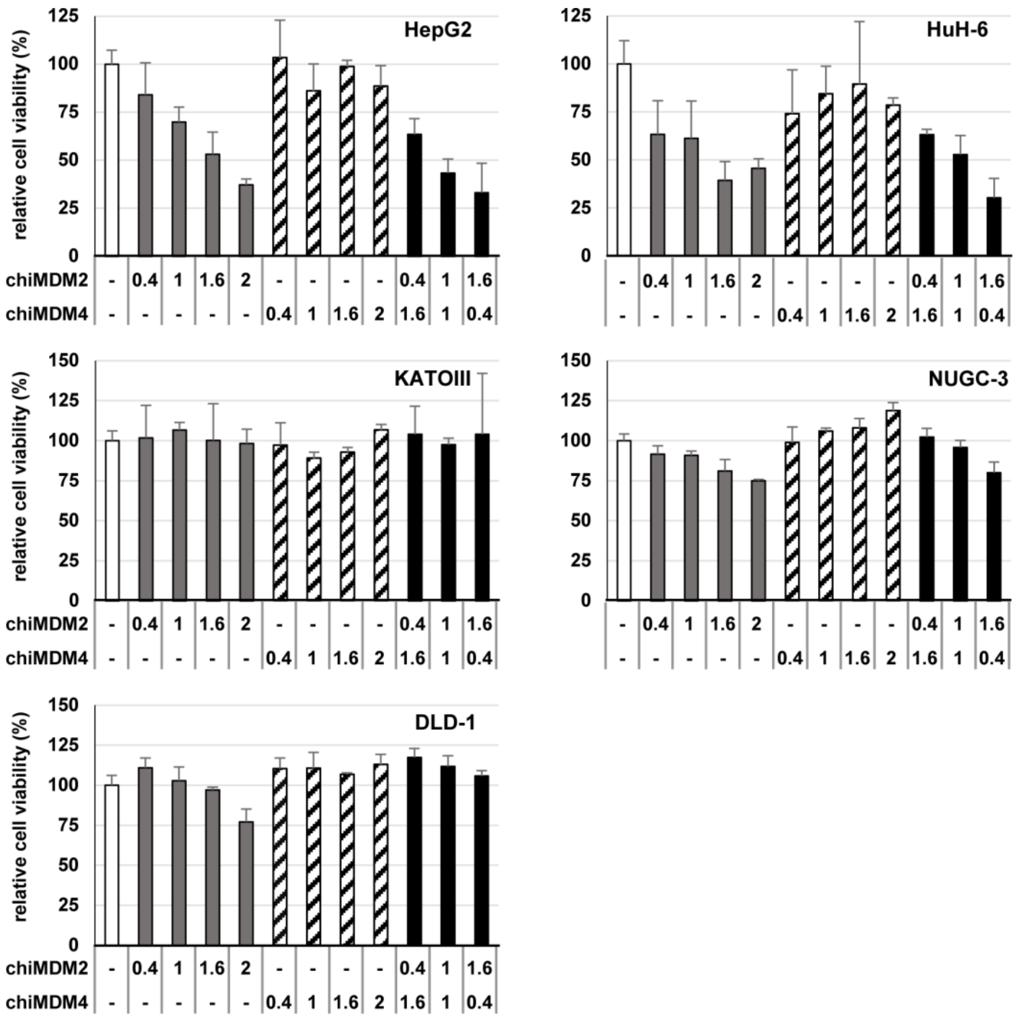

Supplementary Figure 5: Evaluation of combined knockdown of *MDM2* and *MDM4* on the growth of wtTP53 cells with low MDM4 expression and mt *TP53* cells. Effects of individual and simultaneous knockdown of *MDM2* and *MDM4* on the cell growth were examined in wtTP53 cells with low MDM4 expression (HepG2 and HuH-6) and mt *TP53* cells (KATOIII, NUGC-3, and DLD-1). Cells were transfected with *MDM4* dsRDC (chiMDM4-452) alone, *MDM2* dsRDC (chiMDM2-1489) alone, or both. The total amount of dsRDCs was adjusted to 2 nM by adding control dsRDC (chiCtrl). Cell viability was determined 5 days after transfection using the WST-8 assay. The number of viable chiCtrl (2 nM) transfected cells was defined as 100% (mean ± SD; n = 3).

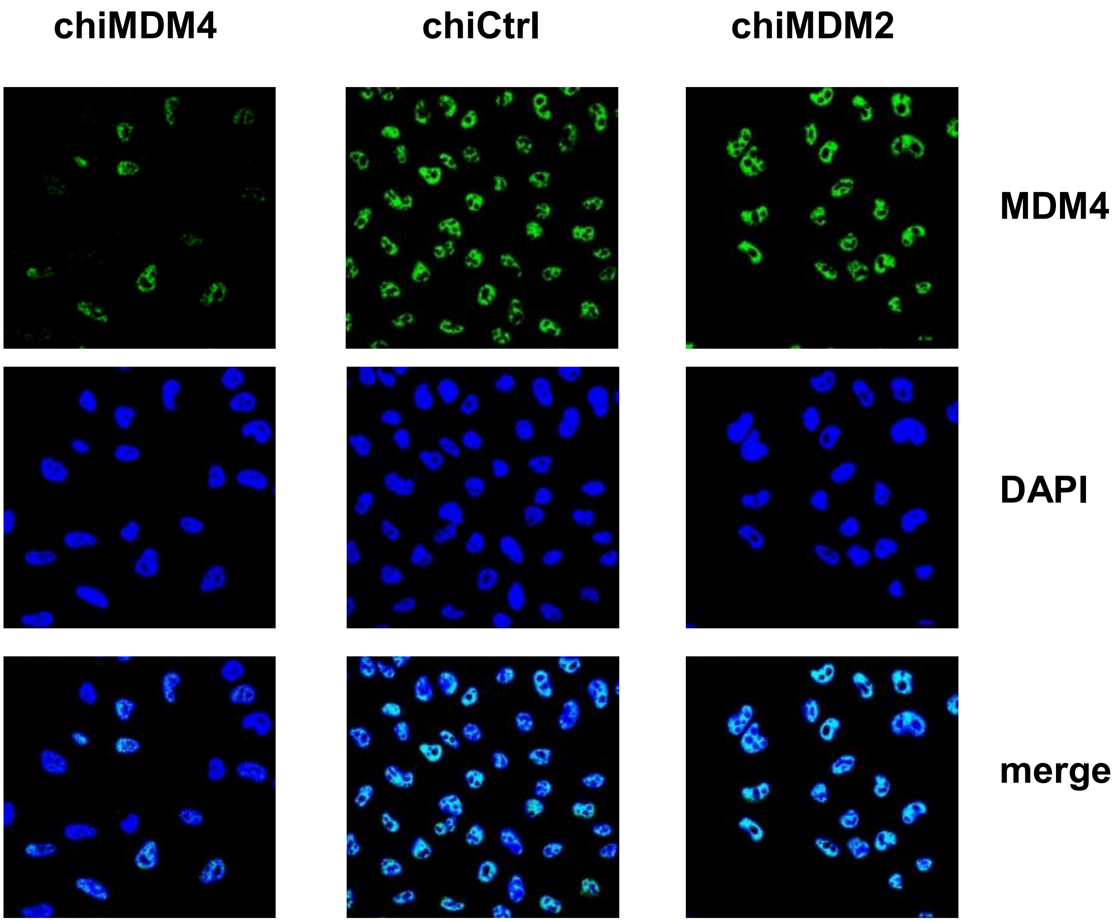

Supplementary Figure 6: Subcellular localization of MDM4 after *MDM2* knockdown. A375 cells were transfected with control dsRDC (chiCtrl), *MDM2* dsRDC (chiMDM2), or *MDM4* dsRDC (chiMDM4) at 1 nM for 24 h. Then, the localization of MDM4 was examined by immunofluorescence. Cellular nuclei were identified by DAPI staining.

Supplementary Table 1: Sequence of siRNAs

siRNAs	Nucleotide position	Passenger strand (5'→3')	Guide strand (5'→3')	References
siMDM2-347	347-369	CCACCUCACAGAUUCCAGCUU	GCUGGAAUCUGUGAGGUGGUU	Linares LK et al.
siMDM2-364	364-386	GCUUCGGAACAAGAGACCCUG	GGGUCUCUUGUUCCGAAGCUG	Yin JQ et al.
siMDM2-373	373-395	CAAGAGACCCUGGUUAGACCA	GUCUAACCAGGGUCUCUUGUU	Linares LK et al.
siMDM2-396	396-418	GCCAUUGCUUUUGAAGUUAUU	UAACUUCAAAAGCAAUGGCUU	Jin Y et al.
siMDM2-636	636-658	UCAGCAGGAAUCAUCGGACUC	GUCCGAUGAUUCCUGCUGAUU	Carrol VA et al.
siMDM2-678	678-700	CAGGUGUCACCUUGAAGGUGG	ACCUUCAAGGUGACACCUGUU	Toh WH et al.
siMDM2-851	851-873	GCCACAAAUCUGAUAGUAUUU	AUACUAUCAGAUUUGUGGCGU	Warburton HE et al.
siMDM2-1644	1644-1666	UGGUUGCAUUGUCCAUGGCAA	GCCAUGGACAAUGCAACCAUU	Kurki S et al.
siMDM2-1713	1713-1735	AAGGAAUAAGCCCUGCCCAGU	UGGGCAGGGCUUAUUCCUUUU	Uchida C et al.
siMDM2-480	480-502	GUAUAUUAUGACUAAACGAUU	UCGUUUAGUCAUAAUAUACUG	
siMDM2-643	643-665	GAAUCAUCGGACUCAGGUACA	UACCUGAGUCCGAUGAUUCCU	
siMDM2-691	691-713	GAAGGUGGGAGUGAUCAAAAG	UUUGAUCACUCCCACCUUCAA	
siMDM2-830	830-852	CUGGUGAACGACAAAGAAAAC	UUUCUUUGUCGUUCACCAGAU	
siMDM2-1068	1068-1090	CUCAGAAGAUUAUAGCCUUAG	AAGGCUAUAAUCUUCUGAGUC	
siMDM2-1274	1274-1296	CCCUUCGUGAGAAUUGGCUUC	AGCCAAUUCUCACGAAGGGCC	
siMDM2-1275	1275-1297	CCUUCGUGAGAAUUGGCUUCC	AAGCCAAUUCUCACGAAGGGC	
siMDM2-1489	1489-1511	CAGCCAUCAACUUCUAGUAGC	UACUAGAAGUUGAUGGCUGAG	
siMDM2-1625	1625-1647	GUCAAGGUCGACCUAAAAAUG	UUUUUAGGUCGACCUUGACAA	
siMDM2-2370	2370-2392	GGCCUAAAUGUCACUUAGUAC	ACUAAGUGACAUUUAGGCCGG	
siMDM2-2381	2381-3003	CACUUAGUACCUUUGAUAUAA	AUAUCAAAGGUACUAAGUGAC	
siMDM2-3412	3412-3434	CCACCAUUUACCCGUAAGACA	UCUUACGGGUAAAUGGUGGCU	
siMDM2-5187	5187-5209	CUCCAAAGGUAAAAGUACUAA	AGUACUUUUACCUUUGGAGGU	
siMDM2-6068	6068-6090	GGUUCUUUAUAGUACACGUGU	ACGUGUACUAUAAAGAACCUA	
siMDM2-6281	6281-6303	GCAGUUGGGAGCCUCCAAUGA	AUUGGAGGCUCCCAACUGCUU	
siMDM2-6393	6393-6415	GUGAUCGUGAAUGGUCUAUAA	AUAGACCAUUCACGAUCACUU	
siMDM2-7414	7414-7436	GUACUCAAAUAUUUAACGUUA	ACGUUAAAUAUUUGAGUACAG	
siMDM4-317	317-339	GGUCAUGCACUAUUUAGGUCA	ACCUAAAUAGUGCAUGACCUC	
siMDM4-347	347-369	GGUGAAGCAACUUUAUGAUCA	AUCAUAAAGUUGCUUCACCAU	

**siMDM4-452**	**452-474**	**CCCUCUCUAUGAUAUGCUAAG**	**UAGCAUAUCAUAGAGAGGGCU**
**siMDM4-525**	**525-547**	**GCUCUCGCACAGGAUCACAGU**	**UGUGAUCCUGUGCGAGAGCGA**
**siMDM4-582**	**582-604**	**GCAGAGGAAAGUUCCACUUCC**	**AAGUGGAACUUUCCUCUGCAC**
**siMDM4-788**	**788-810**	**CAACUAUACACCUAGAAGUAA**	**ACUUCUAGGUGUAUAGUUGCU**
**siMDM4-861**	**861-883**	**GAUACUACAGAUGACUUGUGG**	**ACAAGUCAUCUGUAGUAUCUG**
**siMDM4-1036**	**1036-1058**	**GUGAUGAUACCGAUGUAGAGG**	**UCUACAUCGGUAUCAUCACUU**
**siMDM4-1492**	**1492-1514**	**GACCACGAGACGGGAACAUUA**	**AUGUUCCCGUCUCGUGGUCUU**
**siMDM4-2263**	**2263-2285**	**GAGACUAUAGACUAGCAUAAC**	**UAUGCUAGUCUAUAGUCUCAG**
**siControl**		**GUACCGCACGUCAUUCGUAUC**	**UACGAAUGACGUGCGGUACGU**
**siControl-R**		**CCGUACUAGCCAUUAUGCGUC**	**CGCAUAAUGGCUAGUACGGGU**

Supplementary References

1. Linares LK et al. HdmX stimulates Hdm2-mediated ubiquitination and degradation of p53. Proc Natl Acad Sci USA. 2003; 100: 12009-14
2. Yin JQ et al. siRNA agents inhibit oncogene expression and attenuate human tumor cell growth. J Exp Ther Oncol. 2003; 3: 194-204
3. Jin Y et al. MDM2 promotes p21waf1/cip1 proteasomal turnover independently of ubiquitylation. EMBO J. 2003; 22: 6365-77
4. Carrol VA et al. Regulation of angiogenic factors by HDM2 in renal cell carcinoma. Cancer Res. 2008; 68: 545-52
5. Toh WH et al. Relief of p53-mediated telomerase suppression by p73. J Biol Chem. 2005; 280: 17329-38
6. Warburton HE et al. p53 regulation and function in renal cell carcinoma. Cancer Res. 2005; 65: 6498-503
7. Kurki S et al. Nucleolar protein NPM interacts with HDM2 and protects tumor suppressor protein p53 from HDM2-mediated degradation. Cancer Cell. 2004; 5: 465-75
8. Uchida C et al. Enhanced Mdm2 activity inhibits pRB function via ubiquitin-dependent degradation. EMBO J. 2005; 24: 160-169
